# *Lactobacillus helveticus* SBT2171 Inhibits Lymphocyte Proliferation by Regulation of the JNK Signaling Pathway

**DOI:** 10.1371/journal.pone.0108360

**Published:** 2014-09-30

**Authors:** Tomohiro Hosoya, Fumihiko Sakai, Maya Yamashita, Takuya Shiozaki, Tsutomu Endo, Ken Ukibe, Hiroshi Uenishi, Yukio Kadooka, Tomohiro Moriya, Hisako Nakagawa, Yosuke Nakayama, Tadaaki Miyazaki

**Affiliations:** 1 Milk Science Research Institute, Megmilk Snow Brand Co. LTD., Saitama, Japan; 2 Department of Probiotics Immunology, Institute for Genetic Medicine, Hokkaido University, Hokkaido, Japan; 3 Division of Molecular Immunology, Institute for Genetic Medicine, Hokkaido University, Hokkaido, Japan; Institut national de la santé et de la recherche médicale - Institut Cochin, France

## Abstract

*Lactobacillus helveticus* SBT2171 (LH2171) is a lactic acid bacterium with high protease activity and used in starter cultures in the manufacture of cheese. We recently reported that consumption of cheese manufactured using LH2171 alleviated symptoms of dextran sodium sulfate (DSS)-induced colitis in mice. In this study, we have examined whether LH2171 itself exerts an inhibitory effect on the excessive proliferation of lymphocytes. We found that LH2171 inhibited the proliferation of LPS-stimulated mouse T and B cells, and the human lymphoma cell lines, Jurkat and BJAB. Cell cycle analysis showed an accumulation of LH2171-treated BJAB cells in the G2/M phase. Further, phosphorylation of c-Jun N-terminal kinase (JNK) and c-Jun was reduced by LH2171 in BJAB cells. Subsequently, expression of cell division cycle 2 (CDC2), regulated by the JNK signaling pathway and essential for G2/M phase progression, was inhibited by LH2171. It was also demonstrated that intraperitoneal administration of LH2171 strongly alleviated symptoms of collagen-induced arthritis (CIA) in mice. These findings suggest that LH2171 inhibits the proliferation of lymphocytes through a suppression of the JNK signaling pathway and exerts an immunosuppressive effect *in vivo*.

## Introduction

The immune system is normally maintained by a balance between protective immunity against pathogens and tolerance to self-antigens, and disruption to this balance arising from the abnormal activation and proliferation of lymphocytes causes autoimmune diseases associated with chronic inflammation such as rheumatoid arthritis (RA) or inflammatory bowel disease (IBD). The function of B lymphocytes and T lymphocytes, particularly T helper 17 (Th17) cells that produce interleukin-17 (IL-17), contributes to the pathogenesis of autoimmune diseases [1], [2]. Thus, immunosuppressive agents that suppress excessive proliferation of lymphocytes act as therapeutic drugs for inflammatory autoimmune diseases.

Lactic acid bacteria (LAB) are anaerobic bacteria characterized by the production of lactic acid and used for fermented foods such as dairy products. Recently, some strains of LAB have attracted attention as probiotics, live microorganisms which when administered in adequate amounts confer health benefits on the host. Some probiotic strains of LAB have been shown to contribute to the host immune homeostasis by modulating the innate and adaptive immune system [3]. Further, recent studies have shown that some strains of LAB and its components directly suppress the proliferation of lymphocytes *in vitro* [4], [5], [6]. In the process of activation and proliferation of lymphocytes, intracellular signaling pathways are mediated by mitogen-activated protein kinases (MAPKs) including the c-Jun N-terminal kinase (JNK) [7]. The JNK is activated following its phosphorylation by MAPK kinase 4 (MKK4) and MKK7 and is responsible for the phosphorylation and activation of transcription factor, c-Jun [8], [9]. Phosphorylated and activated c-Jun forms homo or heterodimers with other transcription factor activator protein-1 (AP-1) family members and enhances transcription of a wide variety of target genes [10]. However, whether LAB strains suppress these intracellular signaling pathways to inhibit the proliferation of lymphocytes remains unclear.

*Lactobacillus helveticus* (*L. helveticus*) SBT2171 (LH2171) is a strain exhibiting the highest protease activity among 28 *L. helveticus* strains including a type strain, *L. helveticus* JCM1120^T^ (LH1120T) [11]. Further studies showed that several unique proteolytic enzymes of LH2171 were responsible for its high protease activity [12], [13]. Taking advantage of its high protease activity, LH2171 is used in starter cultures in the manufacture of Gouda-type cheese. We recently reported that consumption of cheese manufactured using LH2171 alleviated symptoms of dextran sodium sulfate (DSS)-induced colitis in mice [14]. This relief of symptoms was associated with decreased production of proinflammatory cytokines, IL-17 and IL-6, by cells isolated from intestinal lymphatic tissue, Peyer's patches. A previous study has further shown that LH2171 suppressed the proliferation of lipopolysaccharide (LPS)-stimulated mouse splenocytes [15]. Although the mechanism through which the cheese manufactured with LH2171 alleviated murine DSS-induced colitis remains unknown, these findings raise the possibility that LH2171 itself may exert a suppressive effect on the excessive activation and proliferation of lymphocytes.

In the present study, we examined the suppressive effect of LH2171 on lymphocyte proliferation and the molecular mechanism involved focusing on the intracellular signaling pathways. We demonstrate that LH2171 inhibits the proliferation of LPS-stimulated mouse T and B cells, and human lymphoma cell lines, Jurkat and BJAB, *in vitro*. It was also established that LH2171 inhibited the cell cycle progression of BJAB cells through the suppression of the JNK signaling pathway. Further, we demonstrate that intraperitoneal administration of heat-killed LH2171 alleviate the symptoms of collagen-induced arthritis (CIA) in mice.

## Materials and Methods

### Bacterial strains

*Lactobacillus helveticus* SBT2171 (LH2171) was isolated by Megmilk Snow Brand (Tokyo, Japan). Two type strains of *Lactobacillus*, *Lactobacillus helveticus* JCM1120^T^ and *Lactobacillus gasseri* JCM1131^T^, were provided by Riken Bioresource Center (Ibaraki, Japan). LAB were inoculated into Lactobacilli MRS broth (BD Biosciences, CA, USA) and cultivated for 16 hours at 37°C. Bacterial cells were harvested by centrifugation at 3,000×g for 10 min. Harvested bacterial cells were washed twice with distilled water and freeze-dried. Freeze-dried bacterial cells were resuspended in phosphate buffered saline (PBS) at 10 mg/mL and killed by heating at 80°C for 30 min.

### Mice

Male C57BL/6N and DBA/1J mice were purchased from Japan SLC (Shizuoka, Japan). All mice were given sterile water and standard chow (Labo MR stock, Nosan corporation, Yokohama, Japan) *ad libitum*. All animal experiments were carried out in accordance with the guidelines of the Bioscience Committee of Hokkaido University and were approved by the Animal Care and Use Committee of Hokkaido University.

### Antibodies and reagents

Pacific Blue-conjugated anti-mouse CD3ε antibody (clone 145-2C11), PE-Cy7-conjugated anti-mouse CD19 antibody (clone 6D5), Fluorescein isothiocyanate (FITC)-conjugated annexin V, Propidium iodide (PI), and 7-amino-actinomycin D (7-AAD) were purchased from BioLegend (CA, USA). Alexa Fluor 488-conjugated anti-mouse CD4 antibody (clone RM4-5) was purchased from BD Biosciences. Rabbit anti-phospho-JNK antibody (clone 81E11), rabbit anti-JNK antibody, rabbit anti-phospho-c-Jun antibody (clone D47G9), rabbit anti-c-Jun antibody (clone 60A8), rabbit anti-cdc2 antibody, rabbit anti-β-actin antibody (clone 13E5), and horseradish peroxidase (HRP)-conjugated anti-rabbit IgG antibody were purchased from Cell signaling technology (MA, USA). Lipopolysaccharide (LPS) was purchased from Sigma-Aldrich (MO, USA). RNase A was purchased from Roche Diagnostics (Mannheim, Germany). SuperKiller TRAIL was purchased from Enzo Life Sciences (NY, USA).

### Isolation of immune cells

Male C57BL/6N (7-10-week-old) mice were sacrificed by isoflurane (Intervet, Tokyo, Japan) inhalation, and spleens were harvested. Spleens were mechanically disrupted in RPMI-1640 medium supplemented with 10% fetal bovine serum (FBS), 10 mM HEPES buffer, 2 mM L-glutamine, 100 U/mL penicillin, 100 µg/mL Streptomycin, and 0.05 mM 2-mercaptoethanol. Splenocyte suspensions were filtered through 70-µm cell strainers (BD Biosciences). Erythrocytes remaining in the splenocyte suspensions were eliminated by RBC lysis buffer (BioLegend). For isolation of mouse T cells and B cells, freshly harvested splenocytes were washed with FACS buffer (PBS containing 1% FBS and 0.05% sodium azide), and incubated with Pacific Blue-conjugated anti-mouse CD3ε antibody, Alexa Fluor-conjugated 488 anti-mouse CD4 antibody, and PE-Cy7-conjugated anti-mouse CD19 antibody for 30 min at 4°C. Cells were washed twice with FACS buffer, and T cells (CD3^+^ CD4^+^ cells) and B cells (CD19^+^ cells) were sorted on a FACSAria II cell sorter (BD Biosciences). Viable cell counts were performed by trypan blue dye exclusion assay.

### Cell culture

Mouse splenocyte, T cells, and B cells were cultured in RPMI-1640 medium supplemented with 10% FBS, 10 mM HEPES buffer, 2 mM L-glutamine, 100 U/mL penicillin, 100 µg/mL Streptomycin, and 0.05 mM 2-mercaptoethanol at 37°C in 5% CO_2_. Jurkat, BJAB, and RAW264.7 cells were cultured in RPMI-1640 medium containing 10% FBS, 100 U/mL penicillin, 100 µg/mL Streptomycin, and 0.05 mM 2-mercaptoethanol at 37°C in 5% CO_2_. Caco-2 cells were cultured in Dulbecco's modified eagle medium containing 10% FBS, 100 U/mL penicillin, 100 µg/mL Streptomycin, and 1% nonessential amino acids at 37°C in 5% CO2.

### Cell proliferation assay

Mouse splenocytes (5×10^4^ cells/well) were cultured with LPS (10 µg/mL) in the presence or absence of lactic acid bacteria (1, 10, 100 µg/mL) in 96-well flat bottom plates (BD Biosciences) for 72 hours. Mouse CD3^+^ CD4^+^ T cells and CD19^+^ B cell (5×10^4^ cells/well) were cultured with LPS (10 µg/mL) in the presence or absence of LH2171 (100 µg/mL) in 96-well flat bottom plates for 96 hours. Jurkat, BJAB (3×10^3^ cells/well), RAW264.7, and Caco-2 cells (1×10^3^ cells/well) were cultured in the presence or absence of lactic acid bacteria (1, 10, 100 µg/mL) in 96-well flat bottom plates for 72 hours. After cultivation, cell proliferation rate was measured using Cell Counting Kit-8 (Dojindo, Kumamoto, Japan) according to the manufacturer's protocol.

### Cell cycle analysis

BJAB cells (5×10^5^ cells/well) were cultured in the presence or absence of LH2171 (100 µg/mL) in 6-well plates (BD Biosciences) for 72 hours. After cultivation, cells were harvested by centrifugation at 1,500×g for 5 min and counted by trypan blue dye exclusion assay. Cells were fixed in 70% ethanol for 30 min at 4°C and washed with PBS. Washed cells were treated with RNase A (100 µg/mL) for 30 min at 37°C and stained with PI (25 µg/mL) for 15 min at 4°C. Cytometric data were acquired using a FACSCanto II flow cytometer (BD Biosciences). The mean percentage of cells in G0/G1, S, and G2/M phases were calculated with FACSDiva software (BD Biosciences).

### Apoptosis assay

BJAB cells (1.5×10^5^ cells/dish) were cultured in the presence or absence of LH2171 (100 µg/mL) in a 60-mm dish (BD Biosciences) for 24 hours. SuperKiller TRAIL (50 ng/ml), recombinant human TNF (tumor necrosis factor) related apoptosis inducing ligand, was used as a positive control. After cultivation, cells were harvested by centrifugation at 1,500×g for 5 min. Cells were washed twice with PBS and stained with FITC-conjugated annexin V and 7-AAD in Annexin V Binding Buffer (BioLegend) for 15 min at room temperature. Cytometric data were acquired using a FACSCanto II flow cytometer and analyzed with FACSDiva software.

### Western blotting

BJAB cells (2.5×10^5^ cells/well) were cultured in the presence or absence of LH2171 (100 µg/mL) in 6-well plates for 72 hours. Mouse splenocytes (2×10^7^ cells/dish) were cultured with LPS (10 µg/mL) in the presence or absence of LH2171 (100 µg/mL) in 100-mm dishes (BD Biosciences) for 72 hours. After cultivation, cells were harvested by centrifugation at 1,500×g for 5 min. Harvested cells were lysed in Radioimmunoprecipitation Assay (RIPA) buffer (50 mM Tris pH 7.4, 150 mM NaCl, 1% NP-40, 0.5% deoxycholic acid, 0.1% SDS) supplemented with cOmplete Mini protease inhibitor cocktail and PhosSTOP phosphatase inhibitor cocktail (Roche Diagnostics) overnight at 4°C with gentle rotation. Residues in these cell lysates were removed by centrifugation. Total protein concentration were determined by BCA protein assay (Thermo Scientific, IL,USA). Equal amounts of proteins were separated by SDS-PAGE (12.5% (w/v) polyacrylamide hand made gels) and transferred to nitrocellulose membranes (Millipore, MA, USA). After transfer, membranes were blocked with 5% BSA in Tris-buffered saline containing 0.1% Tween 20 (TBS-T) for 1 hour at room temperature and incubated with primary antibody overnight at 4°C. Membranes were washed with TBS-T and incubated with HRP-conjugated secondary antibody for 2 hours at room temperature. HRP signals were visualized with Immobilon Western chemiluminescent HRP substrate (Millipore) and LAS-1000 mini image analyzer (Fujifilm, Tokyo, Japan). Band intensities were quantified by ImageJ software (National Institutes of Health, MD, USA).

### Induction of CIA and administration of LH2171

To assess the immunosuppressive effect of LH2171 on collagen-induced arthritis (CIA) in mice, 20 male DBA/1J mice (8-week-old) were divided into 2 groups of 10 mice: (1) CIA mice administered with LH2171 (LH2171 group) and (2) CIA mice administered with PBS (control group). To induce CIA, mice were immunized by intradermal injection at the base of the tail with 100 µg of bovine type II collagen (CII) (Chondrex, WA, USA) which was dissolved in 0.05 M acetic acid and emulsified in Complete Freund's Adjuvant (CFA) (Chondrex, WA, USA). Twenty one days after the first immunization, the mice were immunized with CII emulsified in CFA in the same manner. LH2171 group mice were administered with 1 mg LH2171 by intraperitoneal injection three times a week after the first immunization and daily after the second immunization. With the same frequency, control group mice were administered with the same volume PBS by interperitoneal injection. After the second immunization, mice were observed for measurement of arthritis incidence, disease severity, paw thickness, and body weight. Disease severity of arthritis in each paw was scored on a 0–4 scale, as described by Kanayama *et al*. [16]. In brief: 0, normal; 1, focal slight swelling and/or redness in one digit; 2, moderate swelling and erythema of > 2 digits; 3, marked swelling and erythema of the limb; and 4, maximal swelling, erythema, deformity, and/or ankylosis. The maximum possible score for each mouse was 16.

### Statistical analyis

Data are expressed as a mean ± standard error of the mean (SEM). Unless otherwise noted, statistical evaluations were performed by one-way ANOVA and Tukey-Kramer's test for multiple comparisons or Student's *t*-test for single comparisons. *P*-values < 0.05 were considered statistically significant. All analyses were performed using StatView version 5.0 software (SAS institute, NC, USA).

## Results

### LH2171 inhibits the proliferation of lymphocytes

**Figure 1:**
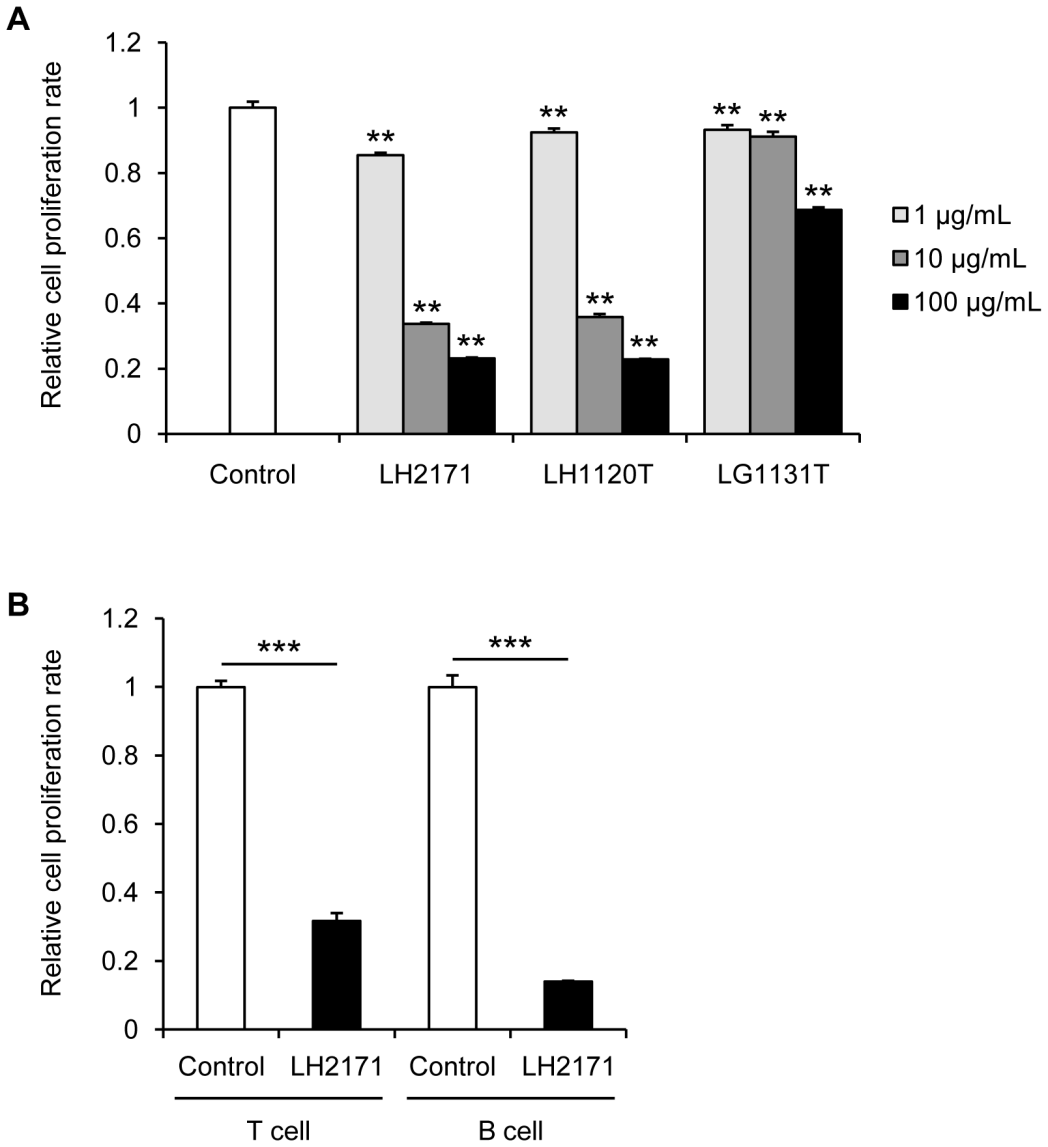
LH2171 suppressed the proliferation of mouse and human immune cells. Splenocytes from C57BL/6N mice (A) were cultured with LPS (10 µg/mL) in the presence or absence of *Lactobacillus helveticus* SBT2171 (LH2171), *Lactobacillus helveticus* JCM1120^T^ (LH1120T), and *Lactobacillus gasseri* JCM1131^T^ (LG1131T) for 72 hours. CD3^+^ CD4^+^ T cells and CD19^+^ B cell (B) isolated from splenocytes of C57BL/6N mice were cultured with LPS (10 µg/mL) in the presence or absence of LH2171 (100 µg/mL) for 96 hours. After cultivation, the cell proliferation rate was measured using a Cell Counting Kit-8 (Dojindo, Kumamoto, Japan). Data are expressed as means ± SEM (n = 3) and compared with the control (**P<0.01, ***P<0.001) by Tukey-Kramer's test (A) and Student's *t*-test (B).

To evaluate the *in vitro* immunosuppressive effect of LH2171, we examined the effect of LH2171 and two types of strains of *Lactobacillus*, *L. helveticus* JCM1120^T^ (LH1120T) and *Lactobacillus gasseri* JCM1131^T^ (LG1131T), on the proliferation of mouse splenocytes. In this assay, splenocytes from C57BL/6N mice were cultured with LPS in the presence or absence of LAB for 72 hours. As shown in Figure 1A, LH2171 treatment significantly inhibited the proliferation of mouse splenocytes in a dose-dependent manner. The inhibitory effect of LH2171 on the proliferation was similar to that of LH1120T and stronger than that of LG1131T (Figure 1A). These results suggest that a strong anti-proliferative effect on the splenocytes is a common feature of *L. helveticus* species.

**Figure 2:**
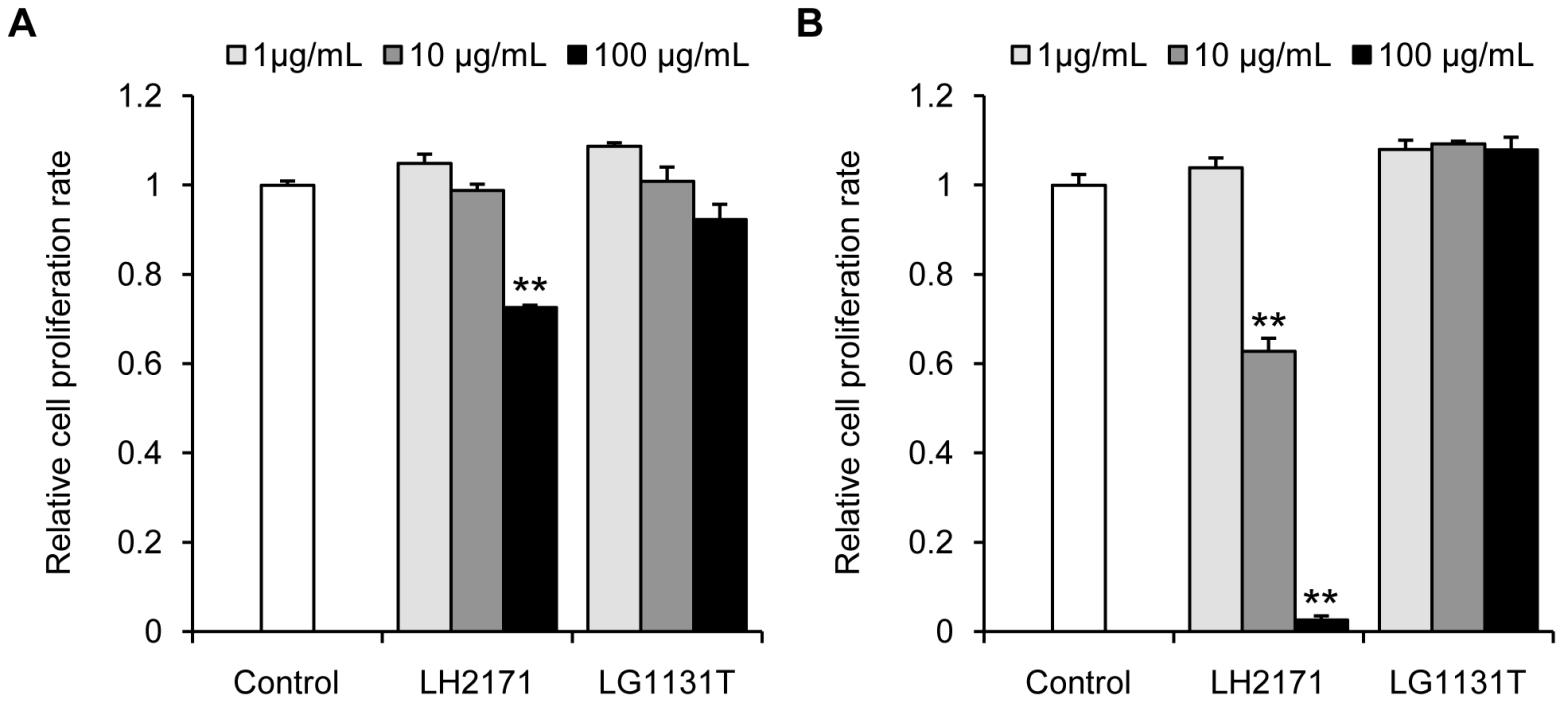
LH2171 suppressed the proliferation of human lymphoma cell lines. Jurkat cells (A) and BJAB cells (B) were cultured in the presence or absence of *Lactobacillus helveticus* SBT2171 (LH2171) and *Lactobacillus gasseri* JCM1131^T^ (LG1131T) for 72 hours. After cultivation, the cell proliferation rate was measured using a Cell Counting Kit-8 (Dojindo, Kumamoto, Japan). Data are expressed as means ± SEM (n = 3) and compared with the control (**P<0.01) by Tukey-Kramer's test.

In order to examine whether LH2171 directly inhibits the proliferation of T and B lymphocytes without antigen presenting cells such as dendritic cells and macrophages. CD3^+^ CD4^+^ T cells and CD19^+^ B cells were isolated from the splenocytes of C57BL/6N mice and cultured with LPS in the presence or absence of LH2171 for 96 hours. The LH2171 treatment significantly inhibited the proliferation of both the CD3^+^ CD4^+^ T cells as well as the CD19^+^ B cells (Figure 1B). Further, we examined whether LH2171 inhibits the proliferation of human T and B lymphocytes using lymphoma cell lines. Human T cell lymphoma Jurkat cells and B cell lymphoma BJAB cells were cultured in the presence or absence of LH2171 and LG1131T for 72 hours. The LH2171 treatment significantly inhibited the proliferation of both Jurkat and BJAB cells, whereas LH1131T treatment did not affect the proliferation of either Jurkat or BJAB cells (Figure 2A, B). These results suggest that LH2171 directly inhibited the proliferation of lymphocytes without antigen presenting cells such as dendritic cells and macrophages. BJAB cells were more sensitive to the anti-proliferative effect of LH2171 than Jurkat cells (Figure 2A, B). To evaluate the sensitivity of other cell lines to LH2171, we examined whether LH2171 inhibits the proliferation of mouse macrophage-like cell line, RAW264.7 and human colon carcinoma cell line, Caco-2. The LH2171 treatment significantly inhibited the proliferation of both RAW264.7 and Caco-2 cells (Figure S1A, B). RAW264.7 cells were more sensitive to the anti-proliferative effect of LH2171 than Caco-2 cells. These results suggest that sensitivity to the anti-proliferative effect of LH2171 varied depending on the types of cell lines.

### LH2171 inhibits the cell cycle progression in BJAB cells

**Figure 3:**
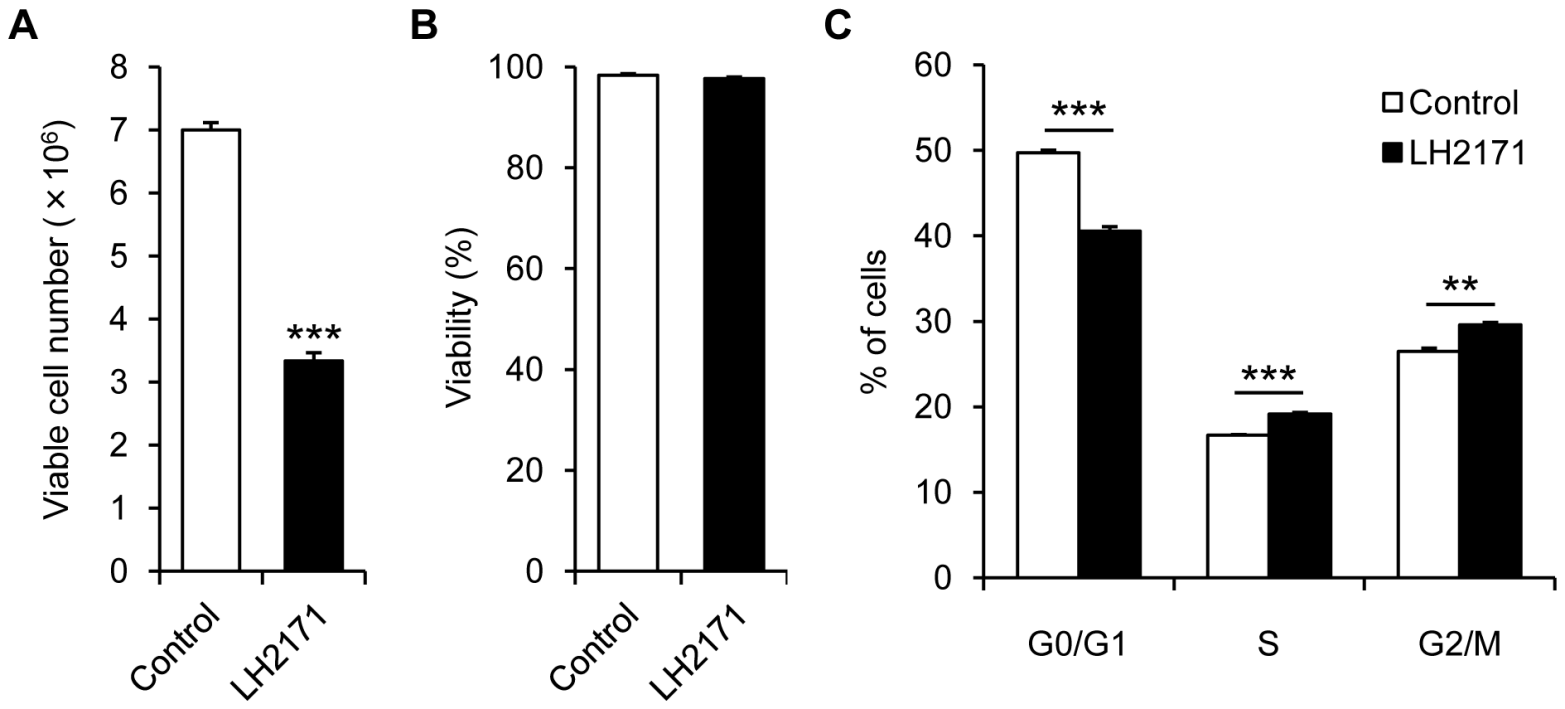
LH2171 inhibited the cell cycle progression in BJAB cells. BJAB cells were cultured in the presence or absence of *Lactobacillus helveticus* SBT2171 (LH2171) (100 µg/mL) for 72 hours. After cultivation, viable cell number (A) and viability (B) were calculated by trypan blue dye exclusion assay, and cell cycle (C) was analyzed by propidium iodide (PI) staining. Data are expressed as means ± SEM (n = 3) and compared with the control (**P<0.01, ***P<0.001) by Student's *t*-test.

As LH2171 strongly inhibited the proliferation of BJAB cells, we examined the mechanism of the anti-proliferative effect of LH2171 using BJAB cells by investigating whether LH2171 exerts a cytotoxic activity or induces apoptosis in BJAB cells. BJAB cells were cultured in the presence or absence of LH2171 for 72 hours, and the viable cell number and viability were determined by a trypan blue dye exclusion assay. The viable cell number of BJAB cells was significantly decreased by the LH2171 treatment (Figure 3A), whereas the viability of the BJAB cells did not change significantly by the LH2171 treatment (Figure 3B). These results suggest that the LH2171 treatment did not increase dead cell numbers of BJAB cells. In annexin V assays, the LH2171 treatment did not increase the number of early apoptotic cells (Annexin V^+^ 7-AAD^−^ cells) and late apoptotic and necrotic cells (Annexin V^+^ 7-AAD^+^ cells), while the SuperKiller TRAIL, recombinant human TNF (tumor necrosis factor) related apoptosis inducing ligand, effectively induced apoptosis in BJAB cells (Figure S2). These results suggest that the anti-proliferative effect of LH2171 was not due to induction of apoptosis.

To investigate whether LH2171 affected the cell cycle progression of BJAB cells, these cells were cultured in the presence or absence of LH2171 for 72 hours, and cellular DNA was stained by PI, then the ratios of cells in the G0/G1, S, and G2/M phases were evaluated by flow cytometry. The LH2171 treatment significantly decreased the ratio of G0/G1 phase cells and increased the ratio of S and G2/M phase cells (Figure 3C). These results suggest that LH2171 inhibited the cell cycle progression of the BJAB cells.

### LH2171 reduces CDC2 expression through the suppression of the c-Jun N-terminal kinase (JNK) signaling pathway in BJAB cells

**Figure 4:**
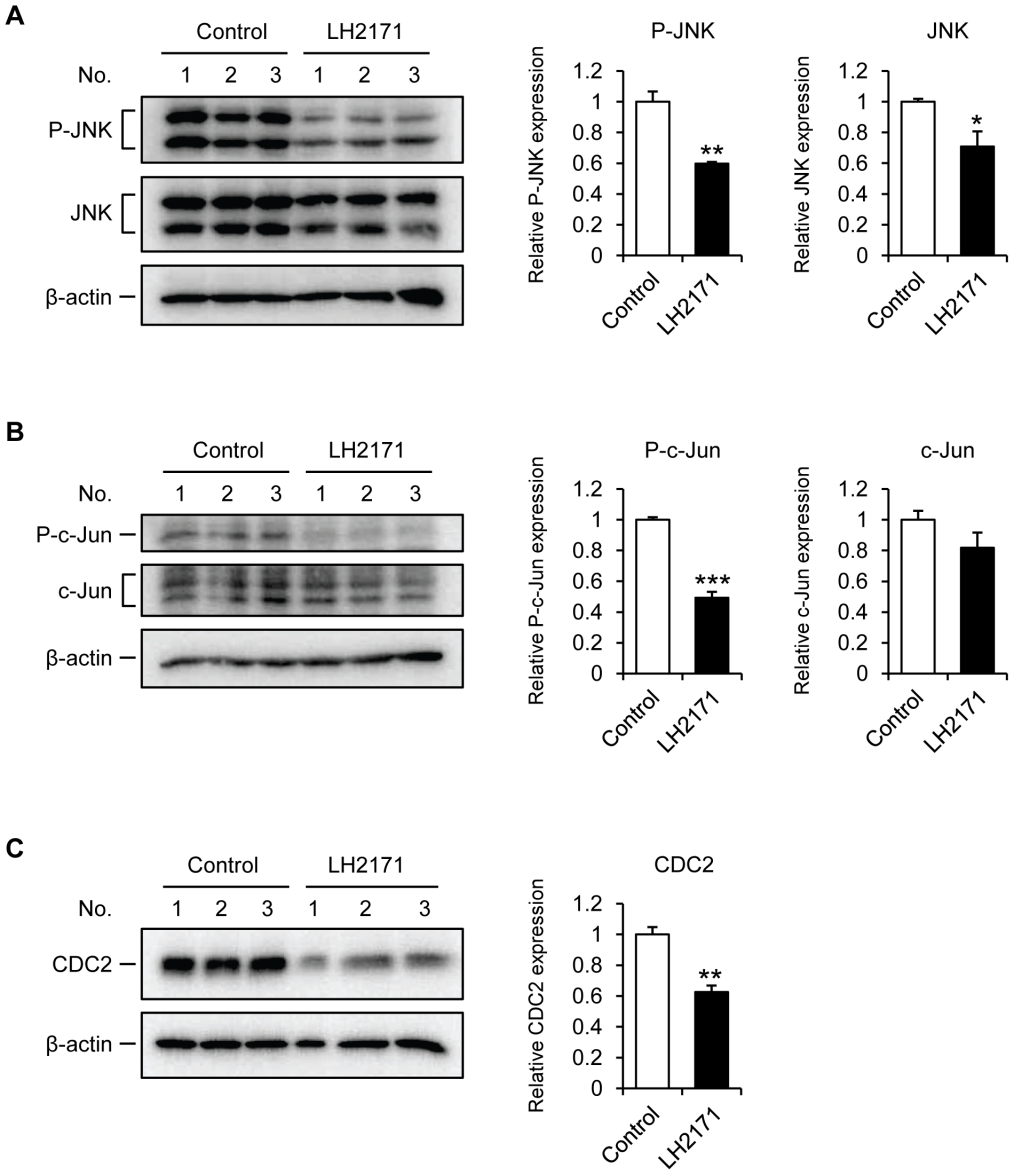
LH2171 reduced CDC2 expression through the suppression of c-Jun N-terminal kinase (JNK) signaling pathway in BJAB cells. BJAB cells were cultured in the presence or absence of *Lactobacillus helveticus* SBT2171 (LH2171) (100 µg/mL) for 72 hours. The cell lysates were analyzed by western blotting to detect the expression of phosphorylated and total protein of JNK (A), c-Jun (B), and CDC2 (C) in triplicate (indicated as 1–3 on the gels). Relative expression of each protein normalized by β-actin expression was quantitated. Data are expressed as means ± SEM (n = 3) and compared with the control (*P<0.05, **P<0.01, ***P<0.001) by Student's t-test.

A previous study has demonstrated that the constitutive activation of the JNK signaling pathway is required for the proliferation of mouse and human B lymphoma cell lines including BJAB [17]. We investigated whether the JNK signaling pathway is involved in the LH2171-mediated inhibition of BJAB cells proliferation: by culturing BJAB cells in the presence or absence of LH2171 for 72 hours, and determining the level of expression and degree of phosphorylation of both JNK and c-Jun by western blotting. As shown previously [17], constitutive expression of phosphorylated JNK and JNK was detected in the BJAB cells and the expression was significantly decreased by the LH2171 treatment (Figure 4A). The expression of phosphorylated c-Jun was also significantly decreased by the LH2171 treatment, whereas the expression of total c-Jun was not affected by LH2171 treatment (Figure 4B). These findings indicate that LH2171 suppressed the activation of the JNK-c-Jun signaling pathway in BJAB cells.

It has been shown that the JNK-cJun signaling pathway regulates the expression of cell division cycle 2 (CDC2), which is essential for the G2/M phase progression [18], [19], and that a loss of c-Jun results in reduced CDC2 expression, cell cycle arrest in the G2/M phase, and impaired cell proliferation [18]. As the cell cycle analysis showed an accumulation of LH2171-treated BJAB cells in the G2/M phase (Figure 3C), we next investigated whether CDC2 is involved in the LH2171-mediated inhibition of the cell cycle progression in BJAB cells. After cultivation for 72 hours, LH2171 treatment significantly decreased the CDC2 expression in BJAB cells (Figure 4C). These findings suggested that LH2171 reduced the CDC2 expression through suppression of the JNK-c-Jun signaling pathway in BJAB cells.

### LH2171 alleviates collagen-induced arthritis (CIA) in mice

**Figure 5:**
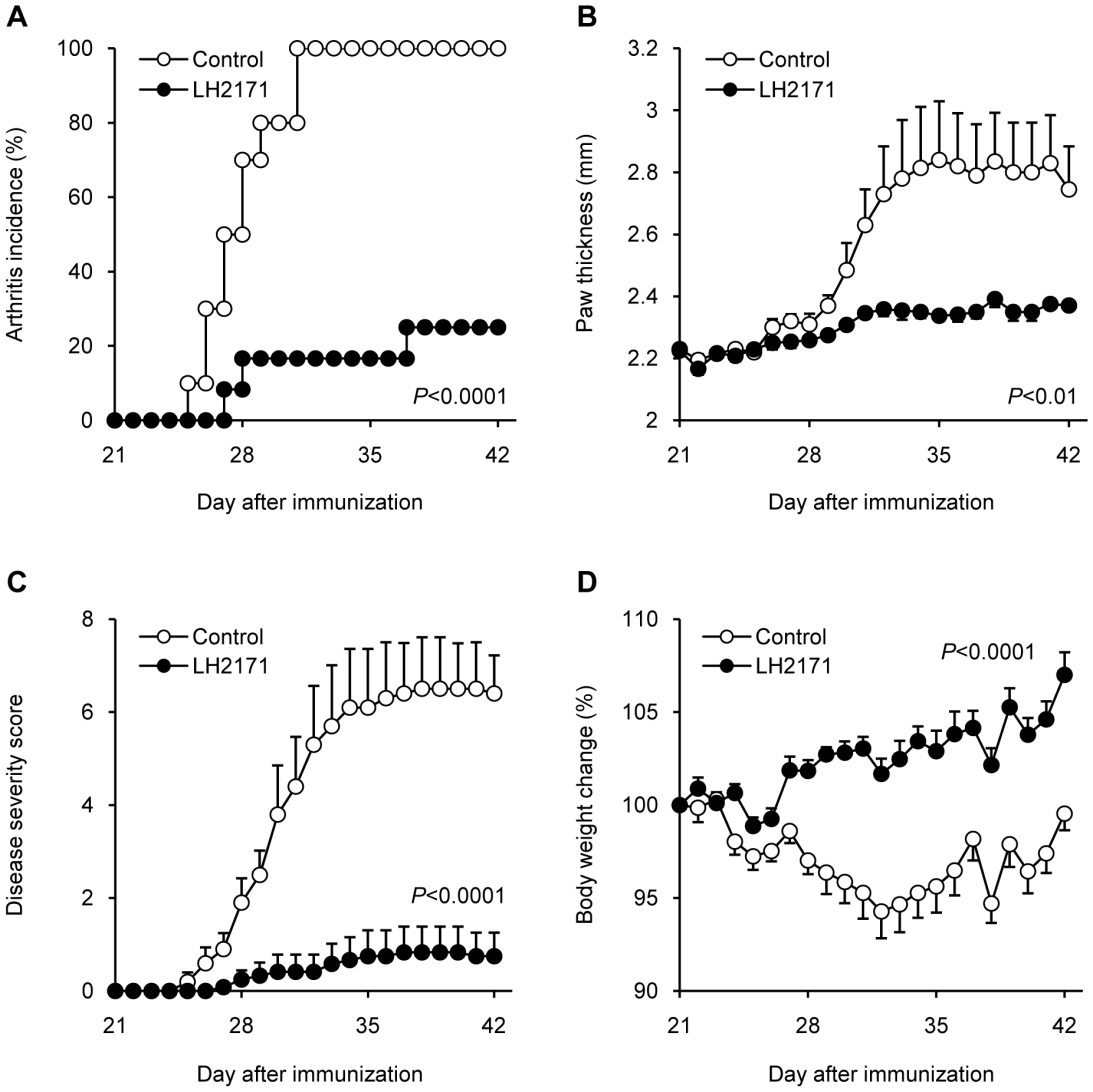
LH2171 alleviated collagen-induced arthritis (CIA) in mice. To induce arthritis, DBA/1J mice (8-week-old) were immunized with an emulsion of 100 µg of bovine type II collagen (CII) which was dissolved in 0.05 M acetic acid and emulsified in Complete Freund's Adjuvant (CFA), and with repeated immunization after 21 days. LH2171 group mice were administered with 1 mg heat-killed *Lactobacillus helveticus* SBT2171 (LH2171) by the intraperitoneal injection three times a week after the first immunization and daily after the second immunization. The control group mice were administered PBS with the same frequency. After the second immunization, mice were observed for measurement of arthritis incidence (A), disease severity score (B), paw thickness (C), and body weight change (D). Data are expressed as means ± SEM (n = 0) and compared with the control group by log-rank test (A) and two-way repeated measures ANOVA (B, C, D).

To evaluate the *in vivo* immunosuppressive effect of LH2171, we examined its effect on collagen-induced arthritis (CIA) in mice. DBA/1J mice were immunized with an emulsion of bovine CII and CFA, and again immunized after 21 days. The mice were administered heat-killed LH2171, or PBS as a control, by intraperitoneal injections three times a week after the first immunization and daily after the second immunization. The arthritis incidence of the LH2171-administrated mice was significantly decreased compared with the control mice (Figure 5A). From 5 days after the second immunization, the paw thickness and disease severity score of the control mice increased gradually to reach a plateau. In contrast, these scores of the LH2171-administrated mice were significantly decreased compared with that of control mice (Figure 5B, C). The body weight loss associated with the arthritis severity was significantly reduced in the LH2171-administrated mice compared with that in the control mice (Figure 5D). Further, the serum CII-specific IgG levels of the LH2171-administrated mice were significantly decreased compared with that of the control mice (data not shown).

## Discussion

The present study demonstrates that LH2171 inhibited the proliferation of LPS-stimulated mouse T and B cells, and the human lymphoma cell lines, Jurkat and BJAB, *in vitro*. We also found that LH2171 inhibited the cell cycle progression of BJAB cells through a suppression of the CDC2 expression and the JNK-c-Jun signaling pathway. Further, we demonstrate that intraperitoneal administration of LH2171 alleviated CIA symptoms in mice.

Intracellular signaling pathways during the activation and proliferation of lymphocytes are mediated by several proteins such as nuclear factor-κ B (NF-κB) and MAPKs including extracellular-signal-regulated kinase (ERK), p38, and JNK [7], [20]. Previous studies have indicated that the ERK and JNK signaling pathways are necessary for T lymphocyte proliferation [21], [22]. Further, the ERK and p38 signaling pathways are necessary for B lymphocyte proliferation [23], [24]. For lymphoma cell lines, constitutive activation of the JNK signaling pathway is necessary for the proliferation of BJAB cells, and SP600125, an anthrapyrazolone inhibitor of JNK, strongly inhibits proliferation of BJAB cells [17]. It has been reported that JNK is activated following phosphorylation by MKK4 and MKK7, and that it is responsible for the phosphorylation of c-Jun [8], [9]. Here, LH2171 reduced the expression of phosphorylated JNK and c-Jun in BJAB cells (Figure 4A, B), and LH2171 also reduced the JNK expression (Figure 4A). Together these findings suggest that LH2171 suppresses JNK activation at the protein level rather than the phosphorylation level.

The JNK signaling pathway is also reported to regulate cell cycle progression via induction and activation of cyclin-dependent kinases (CDKs) and cyclins. CDC2, also known as CDK1, is essential for the progression from the G2 phase to the M phase [19]. It has been shown that CDC2 is a downstream target for the MKK7-JNK-cJun signaling pathway. Further, loss of c-Jun results in reduced CDC2 expression, cell cycle arrest in the G2/M phase, and impaired cell proliferation [18]. In this study, we found an accumulation of LH2171-treated BJAB cells in the G2/M phase (Figure 3C), and LH2171 treatment dramatically decreased CDC2 expression in BJAB cells (Figure 4C). Overall, these findings suggest that LH2171 inhibits the cell cycle progression though reductions in the CDC2 expression, leading to inhibition of proliferation of BJAB cells.

The results here also showed that LH2171 treatment reduced the expression of both CDC2 and phosphorylated c-Jun in LPS-stimulated mouse splenocytes (Figure S3A, B). These findings suggest the possibility that LH2171 inhibits the proliferation of primary lymphocytes via a reduction of the CDC2 expression and a suppression of the JNK signaling pathway. However, LH2171 reduced the c-Jun expression in LPS-stimulated mouse splenocytes (Figure S3A), whereas LH2171 did not reduce the c-Jun expression in BJAB cells (Figure 4B). This difference may arise as the fact that splenocytes include antigen presenting cells such as dendritic cells and macrophages, in contrast BJAB cell is a single lymphoma cell line.

Further study is needed to identify the component contributing to the anti-proliferative effects of LH2171. *Lactobacillus gasseri* OLL2809 and its RNA has been reported to suppress proliferation of mouse CD4^+^ T cells through the MyD88 dependent signaling pathway [5]. Bacterial and viral RNA are recognized by two types of toll-like receptors (TLRs), TLR3 and TLR7. In the present study, treatment of the TLR3 ligand, poly (I:C) and the TLR7 ligand, imiquimod did not suppress the proliferation of BJAB cells (data not shown), although expression of TLR3 and TLR7 in BAJB cells has been reported [25]. These findings suggest the possibility that the TLR signaling pathway is not involved in LH2171-mediated inhibition of BJAB cell proliferation.

We utilized the murine CIA model as an *in vivo* model of inflammatory diseases deriving from dysregulated activation and proliferation of lymphocytes, and evaluated the immunosuppressive effect of LH2171 using this model. The LH2171 treatment inhibited the proliferation of lymphocytes through suppression of the JNK signaling pathway *in vitro*, and also alleviated CIA symptoms in mice. Since B lymphocytes and T lymphocytes, particularly Th17 cells, play a crucial role in the pathogenesis of CIA [1], [26]. It is possible that the inhibition of lymphocyte proliferation by LH2171 contributes to the alleviation of CIA in mice. However, further study is needed to elucidate the mechanism by which LH2171 alleviated the CIA symptoms.

In conclusion, our study shows that LH2171 treatment directly inhibits proliferation of lymphocytes through a suppression of the JNK signaling pathway, and that intraperitoneal administration of LH2171 alleviated CIA symptoms in mice. It is possible that the mechanism by which LH2171 alleviated CIA depends on the suppression of excessive lymphocyte proliferation. The immunosuppressive effect of LH2171 may be beneficial in the treatment of inflammatory autoimmune diseases.

## Supporting Information

Figure S1 **LH2171 suppressed the proliferation of RAW264.7 and Caco-2 cells.** RAW264.7 (A) and Caco-2 cells (B) were cultured in the presence or absence of *Lactobacillus helveticus* SBT2171 (LH2171) and *Lactobacillus gasseri* JCM1131^T^ (LG1131T) for 72 hours. After cultivation, the cell proliferation rate was measured using a Cell Counting Kit-8 (Dojindo, Kumamoto, Japan). Data are expressed as means ± SEM (n = 3) and compared with the control (**P<0.01) by Tukey-Kramer's test.
(TIF)

Figure S2 **LH2171 did not induce apoptosis in BJAB cells.** BJAB cells were cultured in the presence or absence of *Lactobacillus helveticus* SBT2171 (LH2171) (100 µg/mL) for 24 hours. SuperKiller TRAIL (50 ng/ml), recombinant human TNF (tumor necrosis factor) related apoptosis inducing ligand, was used as a positive control. After cultivation, cells were stained with FITC-conjugated annexin V and 7-AAD. Cytometric data were acquired by flow cytometer.
(TIF)

Figure S3 **LH2171 suppressed the expression of c-Jun and CDC2 in LPS-stimulated mouse splenocytes.** Mouse splenocytes were cultured with LPS (10 µg/mL) in the presence or absence of *Lactobacillus helveticus* SBT2171 (LH2171) (100 µg/mL) for 72 hours. The cell lysates were analyzed by western blotting to detect the expression of phosphorylated and total protein of c-Jun (A) and CDC2 (B) in triplicate (indicated as 1–3 on the gels). Relative expression of each protein normalized by β-actin expression was quantitated. Data are expressed as means ± SEM (n = 3) and compared with the control (*P<0.05, **P<0.01, ***P<0.001) by Student's t-test.
(TIF)
