# Supplementary figures and images for: Lactobacillus helveticus SBT2171 Inhibits Lymphocyte Proliferation by Regulation of the JNK Signaling Pathway
Source: PLoS One. 2014 Sep 30;9(9):e108360. doi: 10.1371/journal.pone.0108360 (PMC4182466; doi:10.1371/journal.pone.0108360)

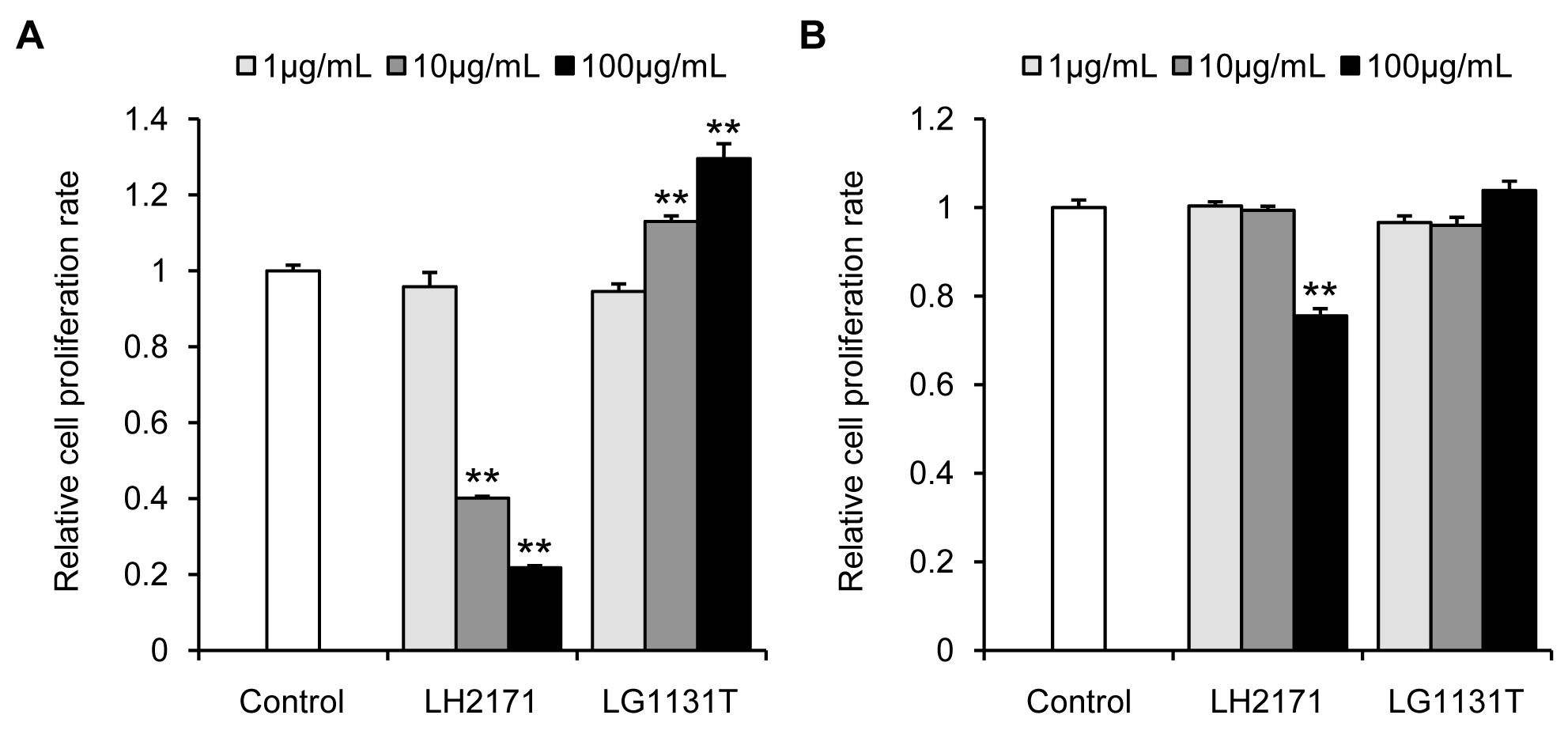

Supplement: Figure S1 — LH2171 suppressed the proliferation of RAW264.7 and Caco-2 cells. RAW264.7 (A) and Caco-2 cells (B) were cultured in the presence or absence of Lactobacillus helveticus SBT2171 (LH2171) and Lactobacillus gasseri JCM1131T (LG1131T) for 72 hours. After cultivation, the cell proliferation rate was measured using a Cell Counting Kit-8 (Dojindo, Kumamoto, Japan). Data are expressed as means ± SEM (n = 3) and compared with the control (**P<0.01) by Tukey-Kramer's test. (TIF) [file pone.0108360.s001.tif]

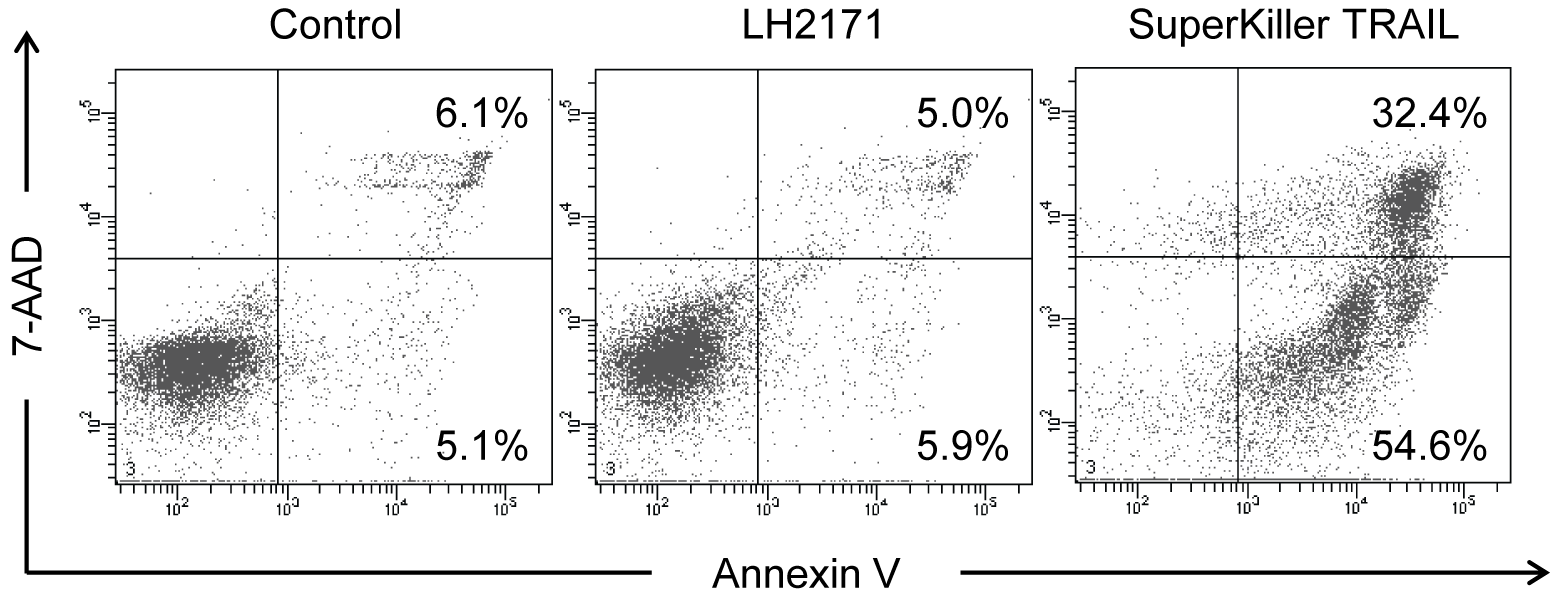

Supplement: Figure S2 — LH2171 did not induce apoptosis in BJAB cells. BJAB cells were cultured in the presence or absence of Lactobacillus helveticus SBT2171 (LH2171) (100 µg/mL) for 24 hours. SuperKiller TRAIL (50 ng/ml), recombinant human TNF (tumor necrosis factor) related apoptosis inducing ligand, was used as a positive control. After cultivation, cells were stained with FITC-conjugated annexin V and 7-AAD. Cytometric data were acquired by flow cytometer. (TIF) [file pone.0108360.s002.tif]

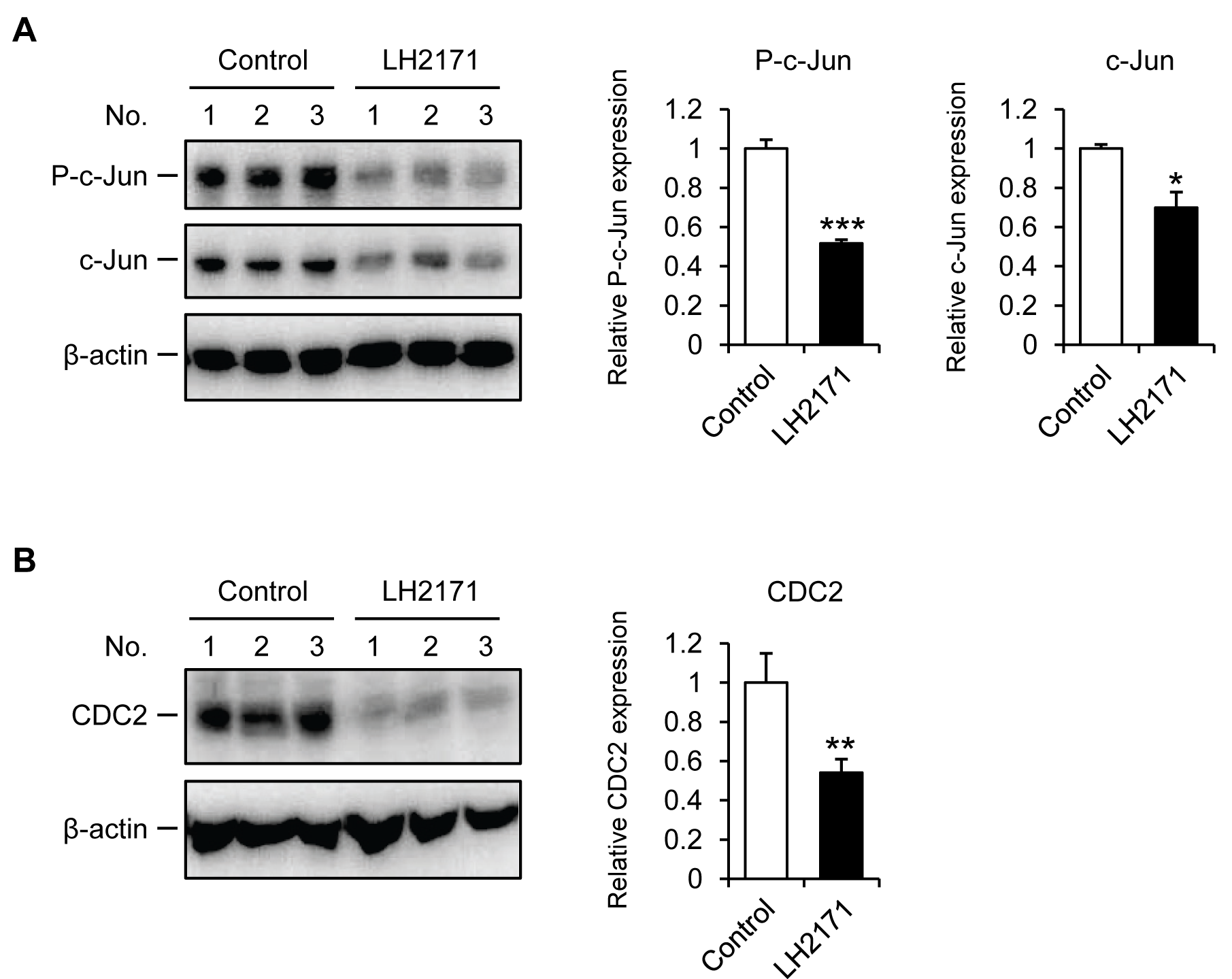

Supplement: Figure S3 — LH2171 suppressed the expression of c-Jun and CDC2 in LPS-stimulated mouse splenocytes. Mouse splenocytes were cultured with LPS (10 µg/mL) in the presence or absence of Lactobacillus helveticus SBT2171 (LH2171) (100 µg/mL) for 72 hours. The cell lysates were analyzed by western blotting to detect the expression of phosphorylated and total protein of c-Jun (A) and CDC2 (B) in triplicate (indicated as 1–3 on the gels). Relative expression of each protein normalized by β-actin expression was quantitated. Data are expressed as means ± SEM (n = 3) and compared with the control (*P<0.05, **P<0.01, ***P<0.001) by Student's t-test. (TIF) [file pone.0108360.s003.tif]
